# A statistical framework for radiation dose estimation with uncertainty quantification from the γ-H2AX assay

**DOI:** 10.1371/journal.pone.0207464

**Published:** 2018-11-28

**Authors:** Jochen Einbeck, Elizabeth A. Ainsbury, Rachel Sales, Stephen Barnard, Felix Kaestle, Manuel Higueras

**Affiliations:** 1 Department of Mathematical Sciences, Durham University, Durham, United Kingdom; 2 Public Health England, Chemical and Environmental Hazards, Chilton, Didcot, United Kingdom; 3 Bundesamt für Strahlenschutz, Fachbereich Strahlenschutz und Gesundheit, Oberschleissheim, Germany; 4 Departamento de Matemáticas y Computación, Universidad de La Rioja, Logroño, La Rioja, Spain; 5 Basque Center for Applied Mathematics, Bilbao, Basque Country, Spain; Northwestern University Feinberg School of Medicine, UNITED STATES

## Abstract

Over the last decade, the *γ*–H2AX focus assay, which exploits the phosphorylation of the H2AX histone following DNA double–strand–breaks, has made considerable progress towards acceptance as a reliable biomarker for exposure to ionizing radiation. While the existing literature has convincingly demonstrated a dose–response effect, and also presented approaches to dose estimation based on appropriately defined calibration curves, a more widespread practical use is still hampered by a certain lack of discussion and agreement on the specific dose–response modelling and uncertainty quantification strategies, as well as by the unavailability of implementations. This manuscript intends to fill these gaps, by stating explicitly the statistical models and techniques required for calibration curve estimation and subsequent dose estimation. Accompanying this article, a web applet has been produced which implements the discussed methods.

## 1 Introduction

For retrospective biological dosimetry following the exposure, or suspected exposure, of individuals to ionizing radiation, a range of viable biomarkers have been identified over the last decades. The most commonly used technique, based on counts of dicentric chromosomes, is well documented by a solid body of literature including the IAEA manual [1], and also well supported by statistical software, including DoseEstimate [2] and CABAS [3]. The dicentric assay enjoys “gold–standard” status due to its good adherence of the aberration counts to the Poisson model, with a stable quadratic dose–response curve which takes very similar shapes for both X– and *γ*–rays [4], and which is robust to inter–individual [1] and inter–laboratory variation [5]. Overdispersion can still arise, for instance from partial body exposure, which can be dealt with through Dolphin’s contaminated Poisson method [6]. Deviation from the Poisson property may also occur under densely ionising radiation or when using automatic scoring procedures, among other causes [7, 8]. The latter types of overdispersion will usually have more serious impact on the uncertainty quantification (UQ) than on the dose estimate itself. The question of how to assess quantitatively the uncertainty related with radiation dose estimates has only started gaining interest very recently, with Bayesian approaches emerging as an interesting device [9, 10]. A comparative study of several UQ techniques including the Bayesian approach was provided in [11].

However, the reason why research on alternative radiation biomarkers continues lies not primarily in the limitations of the dicentric assay mentioned above. A major problem with this assay is the massive resource required to produce a dose estimate. From the moment of exposure, lymphocytes need to be cultured for at least two days until chromosomes reach the metaphase stage of mitosis. Only then can dicentrics be counted, but this process needs to be carried out by skilled and experienced cytogeneticists, and is once more time–intensive. Maznyk et al. [12] estimated that even in ‘triage mode’, where only 50 cells per individual are scored resulting in a detection limit of 0.5Gy [13], the worldwide capacity does not exceed 3000 dose estimates a week, which would clearly be far from sufficient in the case of a large scale radiation accident. Hence, there is a need for alternative biomarkers which allow for higher throughput and arrive at dose estimates, or at least triage classifications, in a quicker, cheaper, and less labour–intensive manner.

Protein–based biomarkers have recently emerged as a potential alternative which possesses all these characteristics. It is known for at least two decades that certain proteins, including the phosphorylated H2AX histone and the p53 binding protein 53PB1, can serve as markers for radiation–induced double strand breaks [14, 15]. The exploration of this property for radiation biodosimetry was discussed from a biomedical viewpoint in Rothkamm and Horn [16]. Further work was subsequently carried out to quantify the dose–response relationship [17, 18] and to assess inter–laboratory variation [13, 19]. In practice the two markers are often co–localized [20]; in this work we concentrate on H2AX only.

Within minutes after exposure of cell cultures to ionizing radiation, the DNA–damage response mechanisms lead to the production of *γ*-H2AX foci, which can be counted through immunofluorescence microscopy in form of little dots (typically, these are red or green, depending on the fluorophore used). Manual, automated, and semi–automated scoring techniques have been investigated for the *γ*–H2AX assay in the literature [13, 19]. Rogakout et al. [14] reported that a maximum 1% of the H2AX proteins become phosphorylated per gray of ionizing radiation. Hence, for low doses, the number of such counts per cell is typically small, and individual foci can usually be well separated under the microscope. A roughly linear relationship between experimental dose and focus counts has been frequently reported [13, 17, 19]; this is physically justified since increasing the dose linearly increases the number of electron tracks and ionisations that produce double–strand breaks [17]. However, when the dose becomes larger than about 3Gy, the H2AX foci have an increasing propensity to overlap, which leads to a saturation effect [19], and may suggest a quadratic rather than linear shape [13]. We will revisit this question in this manuscript from an UQ point of view, but will also settle on linear calibration curves eventually.

Assuming consensus on the *shape* of the dose–response curve, it needs to be recognized that the *parameters* of this curve may vary considerably. Beside the well–understood dependence on time after exposure due to focus loss [17], the curve parameters may also depend on different scoring mechanisms [13], the laboratory [13, 19], the technician who is carrying out the scoring [21], the temperature at exposure [22], shipment [23], as well as the cell type investigated [16, 24]. Furthermore, it has been reported that H2AX focus counts exhibit considerable inter–individual variation [13, 17], which can be partly attributed to covariates like age, smoking, or genetic factors [23]. However, Moquet et al. [19] argued, referring to [24], that this variation only makes a “small contribution” towards the estimation of calibration curve parameters and their uncertainties. Furthermore it has been found that the inter–individual variation operates on a similar scale to the intra–individual variation [22, 25]. We will argue in this article that both sources of sampling variation can be accounted for simultaneously through standard procedures for handling overdispersion.

Given the availability of an adequate calibration curve, it has been recommended that typically 20 (manually scored) or 50 (automated) cells need to be examined from a potentially exposed individual in order to arrive at a dose estimate [19]. This does not require more than 0.1ml of blood which can be conveniently collected through a finger prick sample [26].

Statistical methodology enters into this process twice: Firstly, in order to estimate the calibration curve from laboratory data, and secondly, in order to use this calibration curve to relate the focus count obtained from a potentially exposed individual to the actual dose. While, as outlined above, the biological background literature on the dose–response relationship for the *γ*-H2AX assay is now quite extensive, the development of appropriate statistical methodology has not kept up with this. Even though the statistical techniques required to obtain the dose estimates through the mentioned two steps are relatively basic (weighted least squares and inverse regression, respectively), and of rather similar nature to the methodology used for the dicentric assay [16, 17], the actual challenge resides in the quantification of uncertainty in this process. Ainsbury et al. [11] have recently emphasized that, by ignoring these uncertainties, individuals may get incorrectly triaged with probabilities of up to 50%, with potentially severe consequences for the concerned individual. The computation of these uncertainties is, however, challenging, which has its reasons in (i) the difficulty of the required mathematical and statistical concepts as such (ii) the need to incorporate several different types of uncertainty (iii) the fact that, partly as a consequence of (ii), focus counts are usually strongly overdispersed relative to the mean–variance equality inherent to the Poisson model, and hence standard techniques available for the dicentric assay no longer apply. While it is the case that some methods have been developed to deal with overdispersion in the context of the dicentric assay, such as the consideration of zero–inflated models, [6, 8], it should be stated that the mechanisms which generate overdispersion for the *γ*-H2AX assay are of quite different nature, and also require adjusted methodology to deal with.

It is the purpose of this paper to present and illustrate such methodology, through two previously unpublished H2AX data sets recorded at Public Health England, one of which we will use for calibration curve estimation, and the other one for dose estimation purposes. Both data sets represent homogeneous exposure scenarios, and we do not consider partial exposure in this work. The remainder of the manuscript is organized as follows. In Section 2, we begin with a brief, rather informal, illustration of some features of the calibration data set which motivates qualitatively the modelling decisions that are going to be formally introduced in the two sections which follow: Section 3 describes the statistical modelling techniques used for the estimation of the calibration curve, and Section 4 describes the methodology for dose estimation including uncertainty quantification. Specifically, Sections 4.2 and 4.3 describe how to validate the calibration curve via reference samples, and how to replace the calibration curve by a reference curve if required. Section 5 applies this methodology onto the two mentioned data sets, and also illustrates the web applet that has been developed alongside this article. The manuscript finishes with a Conclusion in Section 6. Since some of the material presented in this paper is inherently technical, a separate ‘tutorial’ has been produced for applied users which illustrates the use of the web applet. The tutorial and the calibration data are available as S1 and S2 Files, respectively.

## 2 Motivation

**Fig 1:**
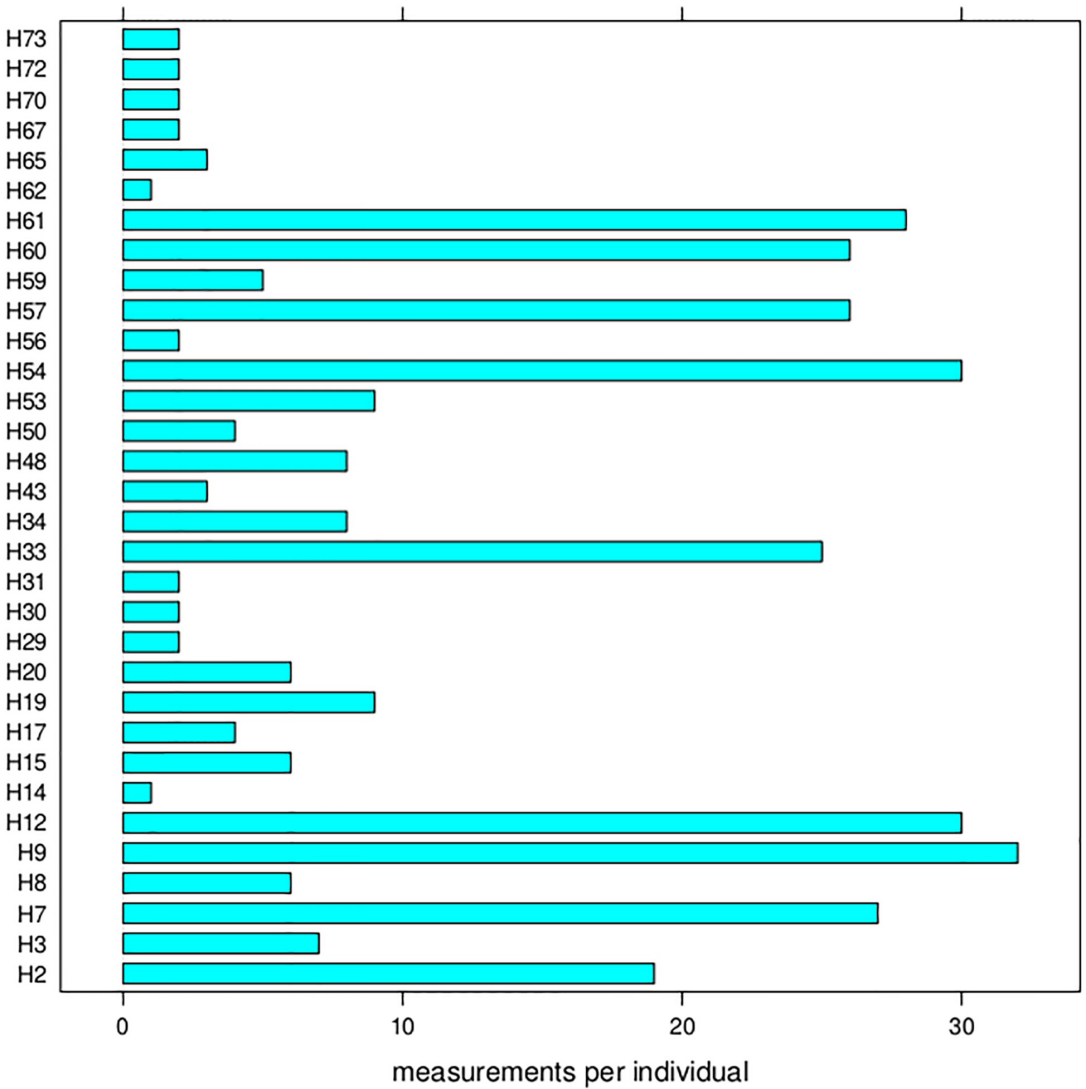
Number of measurements (focus counts over 500 cells) per individual, where the labels on the vertical axis are the anonymized codes of the individuals (donors).

We motivate the developments which are to come through a data set consisting of a total of 339 foci/cell measurements taken from 32 individuals (staff volunteers of Public Health England at Chilton). Heparinized venous blood was taken with written informed consent and the ethical approval of the Berkshire research ethics committee (Ref 09/H0505/87). All samples were anonymized and were ex–vivo irradiated with X–radiation (AGO X-ray limited: 250 kVp, 13 mA at a dose rate of 0.5Gy/min, with 1mm copper and 1mm aluminium filtration). The number of measurements per individual varies between 1 and 32, which is graphically displayed in Fig 1, and all focus counts are out of *n* = 500 cells. The design dose points were taken to be 0Gy, 0.05Gy, 0.1Gy, 0.25Gy, 0.5Gy, 1Gy, and 4Gy, and foci were counted manually, 1h and 24h after exposure. Table 1 gives a breakdown of numbers of measurements per time and dose point. Note that, for most individuals, one only has measurements for a few specific dose and time combinations; for instance for individual H53 for which nine measurements are available, two of them are for 0Gy at 1h, four of them are for 0.5Gy at 1h, and three of them are for 4Gy at 24h. The full data set is made available in .xls and .dat format in S2 File.

**Table 1:** Numbers of measurements (foci counts/cell) of the data introduced in Section 2.

time	design doses (Gy)							Total
	0	0.05	0.1	0.25	0.5	1	4	
1h	56	16	16	16	55	28	0	187
24h	22	16	16	16	19	27	36	152
								339

**Fig 2:**
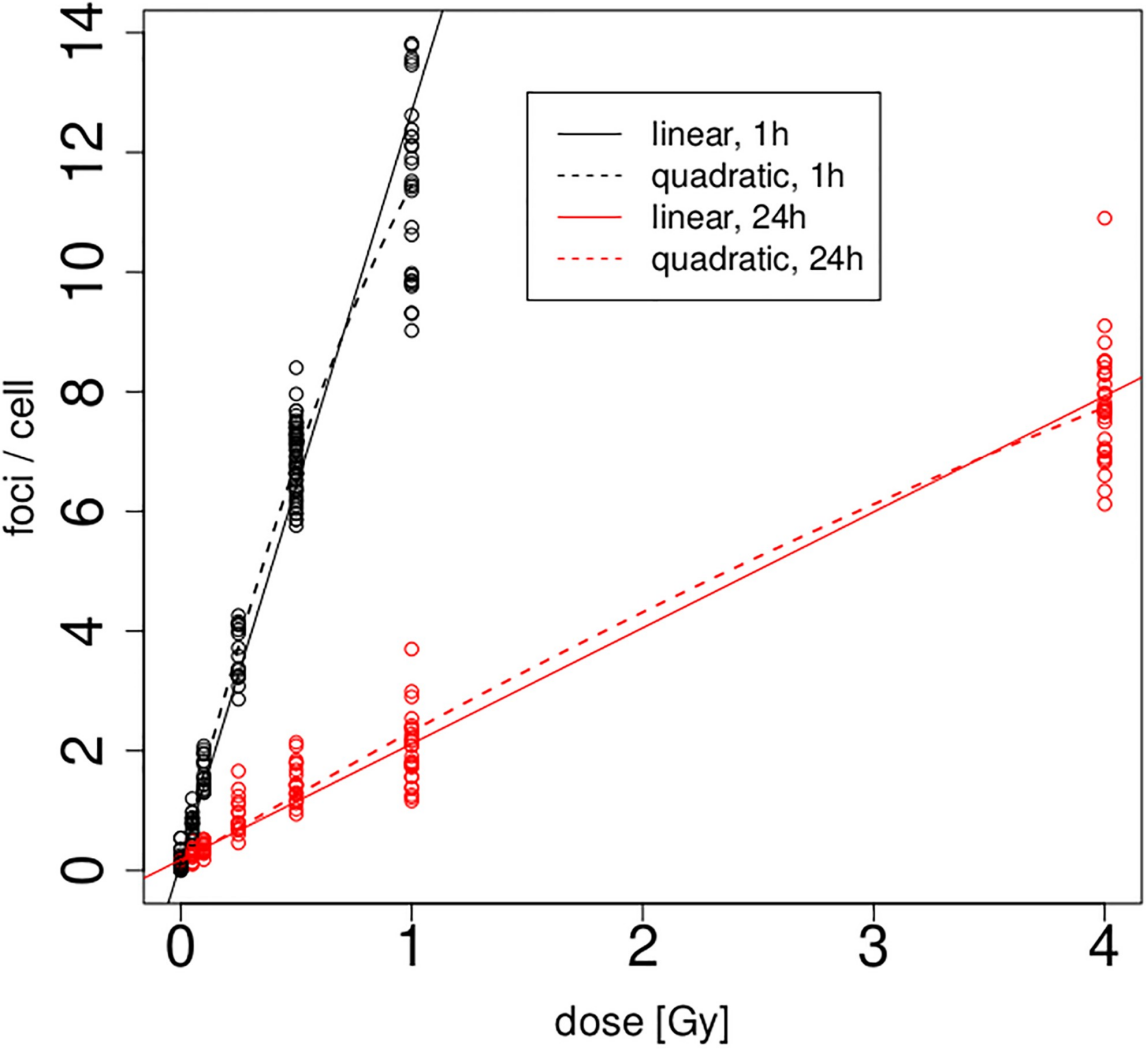
Linear (solid) and quadratic (dashed) calibration curves fitted to 1h and 24h data.

From a modelling point of view, the quantity of interest will be the ‘yield’, hereafter denoted by *y*, which is defined by the number of foci per cell. We fit initially linear and quadratic Poisson regression models of type *E*(*y*) = *A* + *B*dose and *E*(*y*) = *A* + *B*dose + *C*dose^2^, respectively, to the yields, separately for 1h and 24h. The resulting four calibration curves are depicted along with the raw data in Fig 2. It is obvious that the focus counts at 24h are much smaller than at 1h—this is in line with previous literature, which observed that foci disappear at a rate which is consistent with the repair of double–strand breaks [27]. The patterns for both 1h and 24h appear roughly linear, however in each case with a slight saturation effect for large doses which is picked up by the quadratic models. The upper part of Table 2 lists the parameters of all four fitted calibration curves obtained from the Poisson regression, along with standard errors calculated through methodology as explained in the next section. It is worth noting that the saturation effect leads to *negative* quadratic terms in both cases—this is different to calibration curves for dicentric data where the quadratic parameter is usually *positive* (and has a very different physical justification: dicentrics are created through interactions of *pairs* of chromosomes [28]). The current implementation of DoseEstimate [2] would in fact not allow such a negative quadratic term [29].

**Table 2:** Parameter estimates of linear model and quadratic model fitted to the yields (foci/cell). Standard errors are given in round brackets.

separate models		$\hat{A}$	$\hat{B}$	$\hat{C}$
1h	linear	0.131 (0.016)	12.559 (0.161)	
	quadratic	0.112 (0.013)	15.507 (0.368)	-4.169 (0.467)
24h	linear	0.179 (0.021)	1.937 (0.038)	
	quadratic	0.141 (0.021)	2.278 (0.110)	-0.0943 (0.029)
joint model		$\hat{A}$	$\hat{B}$	
1h	linear	0.150 (0.013)	12.518 (0.158)	
24	linear	0.150 (0.013)	1.956 (0.037)	

**Fig 3:**
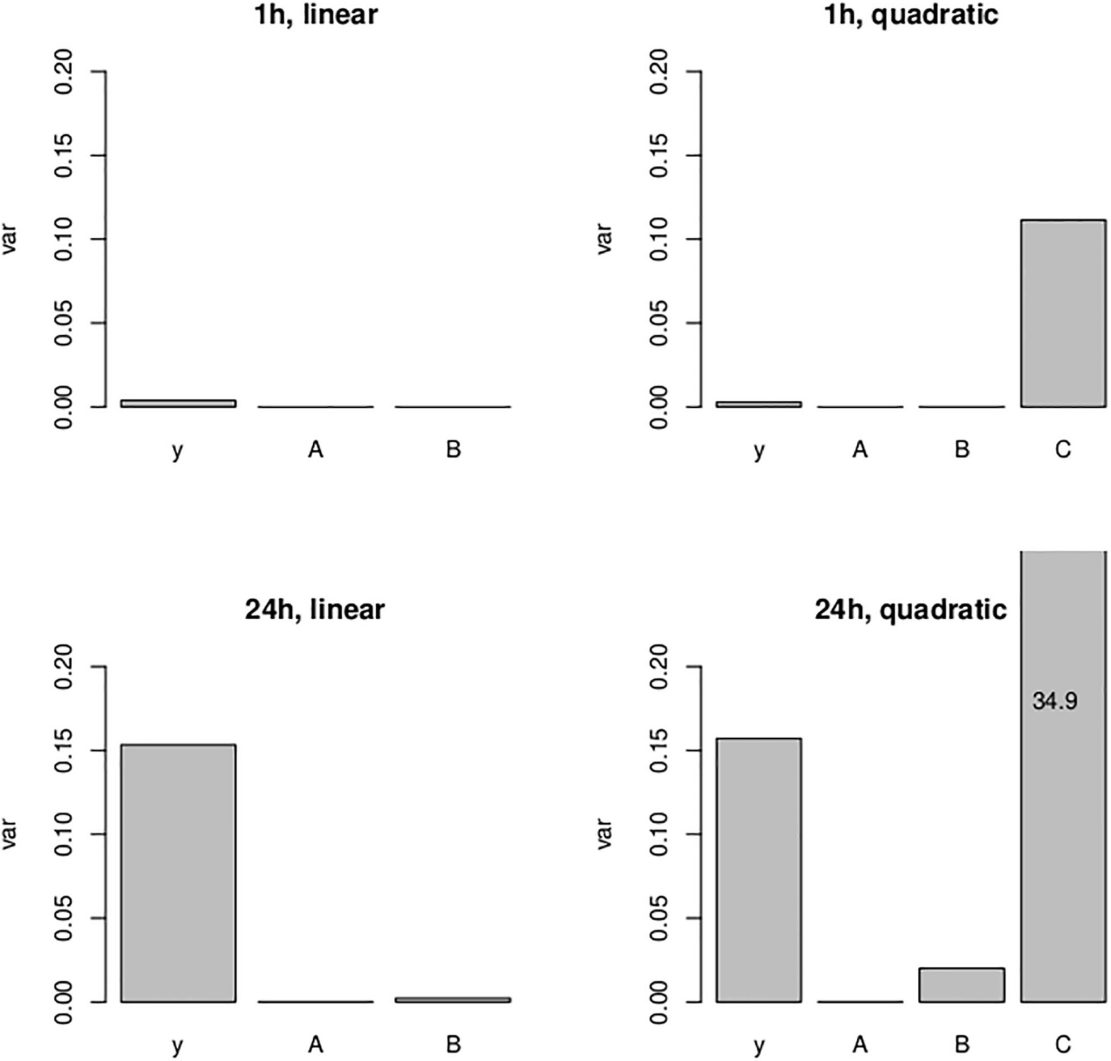
Decomposition of variance of dose estimates for a yield of *y* = 5 foci/cell from *n* = 500 samples, obtained from expansions as in (8). The vertical axis, labelled var, gives the additive contributions of $\hat{A},\hat{B}$ [and where applicable $\hat{C}$], as well as the sampling variance, towards the overall variance of the dose estimate. The contribution of the sampling variance is denoted by *y* on the horizontal axis.

A relevant question is whether the quadratic term is actually needed. The graph in Fig 2 does not give particularly strong evidence to support this. One would expect that the saturation effect, and hence the significance of the quadratic term, is stronger for the 1h than for the 24h data, simply because there are more foci available for the 1h data. It is clear from the parameter estimates and standard errors of the quadratic terms that this is indeed the case. In fact, all p-values of parameters given in Table 2 turn out to be < 10^−9^ except the one for $\hat{C}$ for the 24h data which is equal to 0.00151. While of course even this ‘larger’ p–value still means significance at any reasonable nominal level, it is worth noting that the variance that these quadratic terms contribute can be detrimental to the dose estimation process. Indeed, by expanding the variances of the dose estimates into the contributions of $\hat{A},\hat{B},\hat{C}$ and the sampling variance, one finds that dose estimates can become considerably more imprecise under inclusion of the quadratic term. This is illustrated in Fig 3 for dose estimation after 1h and 24h using the four individual calibration curves from the top part of Table 2. For instance, the top left panel of this figure decomposes the variance of a dose estimate into the uncertainty contributed by the sampling variance, the estimation of $\hat{A}$ and the estimation of $\hat{B}$. Detailed methodology for these calculations will be provided in the next section. One finds that, for the linear model, the contribution by the sampling variance dominates and the uncertainty contributed by the parameter estimates remains negligible compared to that. However, once the quadratic term *C* is included, the variance of this term swamps all other sources of uncertainty and actually grows over all reasonable bounds for the 24h case. Therefore, we will not use quadratic models further in this manuscript.

The role of the intercept parameter, *A*, requires some further discussion. One can consider this parameter as an experiment–specific constant which represents the ‘background yield’, that is the expected yield under zero dose. In the absence of radiation exposure, it is meaningless to speak of ‘time after irradiation’, and hence this constant *A* should be the same for each calibration curve, whatever the time gap considered. In practice, this may be difficult to ensure since calibration curves will often be obtained independently of each other in unrelated experiments, but in our case, where we have 1h and 24 data available obtained under the same experimental conditions, one can fit a joint model

$$\begin{array}{c} E\left(y\right)=a+b_{1}\text{dose}+b_{2}\text{dose}1\left\{\text{time}=24h\right\} \end{array}$$ (1)

so that for the 1h data, one has *A* = *a*, *B* = *b*_1_, and for the 24h data, one finds *A* = *a* and *B* = *b*_1_ + *b*_2_. This leads to the estimates in the bottom part of Table 2, where it is noted that the new, joint, value for $\hat{A}$ lies within two standard errors of the individual estimates. Beside the interpretational advantage, this procedure also leads to smaller parameter standard errors as compared to the separate models. We will use the calibration curves in the bottom part of Table 2 for the remaining analyses in this paper.

**Fig 4:**
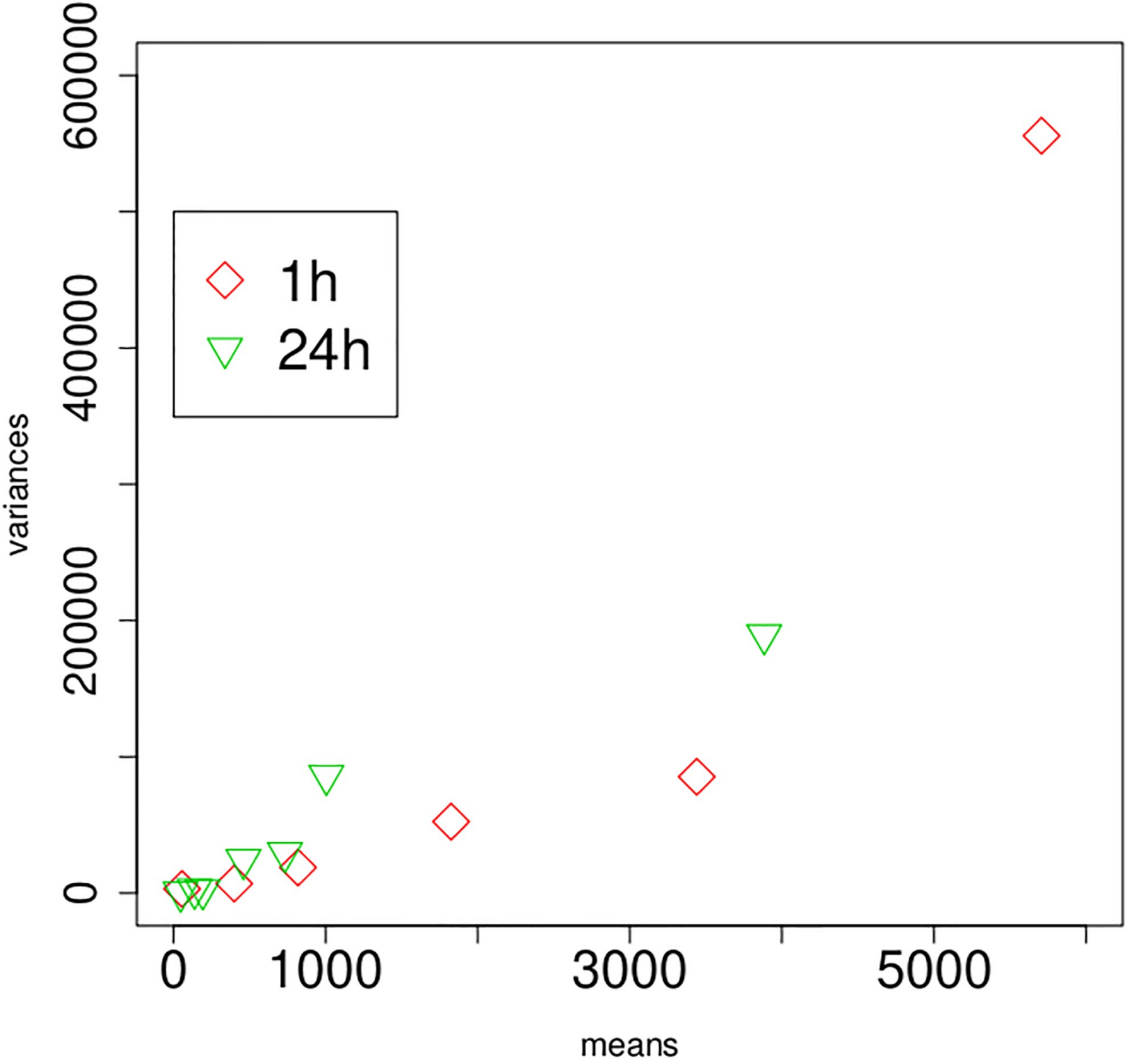
For each dose point, empirical variances vs means for the 1h and 24h data, plotted on the scale of absolute focus counts.

Two questions still need to be discussed. Firstly, as touched upon in the introduction, if the focus counts were Poisson distributed one would assume to observe equality of mean and variance. We therefore computed, for each time and dose point, the means and variances of observed focus counts. The resulting variances are plotted against means in Fig 4. If there was equidispersion, then both the 1h and the 24h data should follow the unity line. It is clear that this is not the case, and that in fact the variances are of a magnitude of 50 to 60 times the mean. This variance/mean ratio, which is also known as the (index of) dispersion, needs to be quantified and taken into account in the modelling, as otherwise uncertainties will be grossly underestimated [29]. Secondly, it has been previously reported in the literature that H2AX focus counts feature stronger inter–individual variation compared to, say, dicentrics [30]. The question is hence whether inter– and intra–individual variation needs to be distinguished in the modelling process. To shed some light on this aspect, we carry out an analysis of deviance on the joint Poisson regression model (1) under inclusion of a factor for donor ID, and we report results in Table 3. We firstly see from this table that a ‘quick–and–dirty’ estimate of the dispersion stands at $\hat{\phi }=17230/\left(339-34\right)\approx 56.49$; in fact the output using R function glm [31] delivers $\hat{\phi }=57.64$ using Eq (5) displayed later in this manuscript. Hence, to assess the significance of the donor terms, the statistic $\Delta \left(\text{Dev}\right)/\hat{\phi }=1786/57.64=30.99$ needs to be compared to $\chi_{31,0.95}^{2}=44.99$, which can be alternatively expressed as a p-value of 0.467. This indicates that the inter–individual variation does not contribute a significant amount of variation, given that variation in dose and time are accounted for. Hence we conclude that the intra-individual variation swamps the inter–individual variation, and both types of variation can be jointly addressed by accounting for overdispersion.

**Table 3:** Analysis of deviance for Poisson linear model involving factors for time (1h/24h) and donor ID (1, …, 32).

Source	df	Deviance explained	Residual deviance
1	1	–	700064
dose	1	273705	426358
time	1	407343	19016
donor	31	1786	17230

Summarizing, in this section we have motivated through a real data set the modelling strategies which shall be implemented later on. Specifically, we suggest that appropriate models for the yield should be linear in dose, should account for the overdispersed count data character of the focus counts, and do not need to distinguish between intra–and inter–individual variation. We provide a mathematical formulation of the required methodology in the next section, and return to the data example in Section 5.1.

## 3 Calibration curve

### 3.1 Modelling and estimation

To fix terms, assume that, in some laboratory, lymphocyte blood samples were obtained from one or more donors according to standard procedures such as outlined in [19]. This blood was divided into *d* parts, and each of these parts exposed to radiation (usually *γ*– or *X*–rays) at different dose levels, *x*_1_, … *x*_*d*_ (typically, these are three to eight values in the range from 0Gy to 4Gy). For each dose level *i* = 1, …, *d*, we examine *n*_*i*_ samples of *n* cells each, and count the total number of foci over all *n* cells within each sample. Common choices are *n* = 200 or *n* = 500. The number *n*_*i*_ of available replicates for each dose level may vary strongly between and within different experiments; in the context of the data from Section 2 the *n*_*i*_ correspond to the values given in Table 1.

Following our reasoning from the previous section, it is secondary for our developments whether these *n*_*i*_ repeated samples are taken from the same individuals, from different individuals, or from blood mixed from several individuals—the intra-individual variation will usually swamp the inter–individual variation. Also, in principle, the number of cells *n* investigated for each sample could be different for each sample or dose level (and hence depend on *i* and *j*), though there is no advantage of doing so, and the calibration data set that we have available for analysis also does not have this feature. Hence, for simplicity of presentation we assume that this number is constant across the full experiment, but the equations below can be adjusted straightforwardly if this is not the case.

For a specific time point after exposure, we denote by *Y*_*ij*_ the *absolute focus count* obtained from the *n* cells under the *j*–th sample (*j* = 1, …, *n*_*i*_) for the *i*–th dose level. It is often more practicable to work with means *y*_*ij*_ = *Y*_*ij*_/*n* rather than absolute numbers, which are also referred to as the *yield* of the experiment. We denote the collection of the $N=\sum_{i=1}^{d}n_{i}$ available yields by $Y=(y_{ij})$, 1 ≤ *i* ≤ *d*, 1 ≤ *j* ≤ *n*_*i*_.

Following our discussion in Section 2, we discard quadratic dose models, and hence relate the yields linearly to dose, via a model

$$\begin{array}{c} \mu_{i}\equiv E\left(y_{ij}|x_{i}\right)=A+Bx_{i} \end{array}$$ (2)

Since the *Y*_*ij*_ = *ny*_*ij*_ are count data, their character needs to be appropriately accounted for in the modelling strategy. A first and natural candidate for their distribution is the Poisson distribution. Rothkamm et al. [13] argue that this assumption is also biologically plausible, namely that “the underlying rationale is that DNA double–strand breaks, and therefore foci, are randomly induced, resulting in a Poisson distribution of foci among the irradiated cell population in case of a whole body exposure”. Hence, under this model the counts *Y*_*ij*_ would be described as

$$\begin{array}{c} Y_{ij}∼\text{Pois}(A\times n+B\times (nx_{i})). \end{array}$$ (3)

Note that obviously (3) entails (2). However, this model also implies the very strong assumption of equidispersion; namely that *μ*_*i*_ = *Var*(*Y*_*ij*_|*x*_*i*_). It is clear from our preliminary considerations that this assumption is not fulfilled for the type of data under consideration, due to considerable intra– and inter–individual variation. Fortunately, there is a simple solution to this problem. The Poisson distribution is a member of the simple exponential family, which possesses two parameters: the so-called natural parameter, *θ*, which relates to the mean, and the dispersion parameter, *ϕ*. In the particular case of the Poisson model, one has *θ* = log(*μ*) where *μ* is the expected response which may depend on covariates as in (2), and *ϕ* is the dispersion parameter which is equal to 1 for the Poisson model. However, it is clear from generalized linear model theory [32] that, if the parameter *ϕ* was allowed to move freely for the Poisson model, the score equations (i.e., the first derivatives of the log–likelihood set equal to 0) for the regression parameters would be a simple multiple of 1/*ϕ*; in our case

$$\begin{array}{c} \frac{1}{\phi }\sum_{i=1}^{d}\sum_{j=1}^{n_{i}}(\begin{array}{c} 1\\ x_{i} \end{array})(Y_{ij}-nA-nBx_{i}))/(A+Bx_{i})=(\begin{array}{c} 0\\ 0 \end{array}). \end{array}$$ (4)

Hence, the parameter *ϕ* can again be cancelled out and so the estimates of the parameters *A* and *B* are unaffected by the unconstrained dispersion. The actual estimates, $\hat{A}$ and $\hat{B}$ are then obtained by an iteratively weighted least squares technique known as Fisher Scoring, details of which are irrelevant for this presentation [32]. Any software routine which can fit generalized linear models can be used for this estimation. The model (3), when equipped with a flexible dispersion parameter, is referred to as a *quasi–Poisson model*, motivated by the fact that a Poisson density with non–unity dispersion does not integrate to 1 and hence the data likelihood is not an actual likelihood. Such a quasi–Poisson model is very useful for our purposes since it, as mentioned, simply reproduces the usual Poisson–based estimates. The standard errors of $\hat{A}$ and $\hat{B}$ do, however, depend on *ϕ*, and therefore the dispersion does need to be estimated.

### 3.2 Dispersion and standard errors

The dispersion associated to model (3) can be estimated consistently [32] via

$$\begin{array}{c} \hat{\phi }=\frac{1}{N-2}\sum_{i=1}^{d}\sum_{j=1}^{n_{i}}\frac{{\left(Y_{ij}-n{\hat{\mu }}_{i}\right)}^{2}}{n{\hat{\mu }}_{i}}=\frac{n}{N-2}\sum_{i=1}^{d}\sum_{j=1}^{n_{i}}\frac{{\left(y_{ij}-{\hat{\mu }}_{i}\right)}^{2}}{{\hat{\mu }}_{i}} \end{array}$$ (5)

where ${\hat{\mu }}_{i}=\hat{A}+\hat{B}x_{i}$. Note that, if additional covariates are used, such as a quadratic term or an indicator variable as in model (1) then the value 2 which is subtracted from *N* in the denominator of (5) needs to be increased by the value 1 for each added model parameter.

The dispersion–corrected standard errors of $\hat{A}$ and $\hat{B}$ can be computed from the uncorrected (Poisson–based) standard errors, say ${\text{SE}}_{P}\left(\hat{A}\right)$ and ${\text{SE}}_{P}\left(\hat{B}\right)$ (which are just the square roots of the diagonal of the inverse Fisher information matrix) via

$$\begin{array}{c} SE\left(\hat{A}\right)=\sqrt{\hat{\phi }}\;{\text{SE}}_{P}\left(\hat{A}\right)\;\;\text{SE}\left(\hat{B}\right)=\sqrt{\hat{\phi }}\;{\text{SE}}_{P}\left(\hat{B}\right). \end{array}$$ (6)

Hence, in practice it is sufficient to fit a simple Poisson regression model (3) to the yields Y and then compute (5) retrospectively to restore the standard errors via (6). We denote the information $C=\{\hat{A},\hat{B},SE\left(\hat{A}\right),SE\left(\hat{B}\right)\}$ as *calibration curve* from now on.

While at some occasion, the value $\hat{\phi }$ will be produced alongside with the calibration curve (and can thus be considered as part of the calibrative information), often this will not be the case, so that the use of a general consensus value may be required. For the calibration curves obtained in Table 2, $\hat{\phi }$ took the values 59.6 (at 1h), 57.5 (at 24h), and 58.7 (joint model). Values of similar magnitude (between 20 and 60) have been obtained in our experiments when using other data sets to calculate the calibration curve, including the one studied in Section 5.1. In many cases the true dispersion of a new sample will be unknown and not estimable or testable, so the consensus value settled on should be a conservative value which represents the ‘worst possible’ overdispersion of focus counts with respect to their Poisson mean. The parameter *ϕ* is, hence, of quite different character to *A* and *B*, which we consider as experiment–specific constants depending on lab, scorer, technology, etc, and which therefore should be determined as precisely as possible. For the remainder of this exposition, we have settled on the value $\hat{\phi }=60$, which leads to virtually identical parameter standard errors as when using the exact dispersion estimates mentioned earlier in this paragraph. Note in this context that a possible misspecification of *ϕ* is somewhat alleviated through the fact that it enters through a square root in all relevant equations. The value $\hat{\phi }=60$ is also used as default value in the web applet that we have produced, but this can be overridden by the user.

## 4 Dose estimation

We consider now the scenario that a given calibration curve C is available. A blood sample has been taken from a potentially exposed individual, and a number n_*_ of cells of this sample have been examined for H2AX foci. One will usually have n_*_ < *n*; it has been argued that for the purpose of triage it is sufficient to have n_*_ ≈ 50 [13, 27]. These n_*_ cells deliver a total focus count Y_*_ and hence a yield y_*_ = Y_*_/n_*_. We summarize this new information by $N=\{y_{*},n_{*}\}$. The task is to arrive at an estimate of dose, x_*_, and its uncertainty, in form of a standard error *SE*(x_*_), using C and N.

The modelling Eq (2) motivates immediately the dose estimator

$$\begin{array}{c} x_{*}=\frac{y_{*}-\hat{A}}{\hat{B}}. \end{array}$$ (7)

We assess the uncertainty attached to this estimate in the following subsection.

### 4.1 Uncertainties

The uncertainties involved in dose estimate (7) can be decomposed via

$$\begin{array}{c} SE^{2}\left(x_{*}\right)={\left(\frac{\partial x_{*}}{\partial \hat{A}}\right)}^{2}SE^{2}\left(\hat{A}\right)+{\left(\frac{\partial x_{*}}{\partial \hat{B}}\right)}^{2}SE^{2}\left(\hat{B}\right)+{\left(\frac{\partial x_{*}}{\partial y_{*}}\right)}^{2}SE^{2}\left(y_{*}\right), \end{array}$$ (8)

which is the MULTIBIODOSE simplification (‘MBD method’) of the delta–method; i.e. it is omitting the covariance terms, the magnitude of which compared to the variance terms can be considered to be “very small” [11]. The partial derivatives in Eq (8) can be worked out to be

$$\begin{array}{ccc} \frac{\partial x_{*}}{\partial \hat{A}} & = & -\frac{1}{\hat{B}} \end{array}$$ (9)

$$\begin{array}{ccc} \frac{\partial x_{*}}{\partial \hat{B}} & = & \frac{y_{*}-\hat{A}}{{\hat{B}}^{2}} \end{array}$$ (10)

$$\begin{array}{ccc} \frac{\partial x_{*}}{\partial y_{*}} & = & \frac{1}{\hat{B}}. \end{array}$$ (11)

That is, in Eq (8), all quantities are immediately known from either C or N, except the sampling error of the yield of the new individual, *SE*(y_*_). To work out this quantity, assume (for a moment) that Y_*_ is a sum of n_*_ Poisson distributed random variables with mean λ_*_ (this does not assume necessarily that this is the same Poisson model as (3), but of course it could be the case). Hence, one can write Var(Y_*_) = n_*_λ_*_, and now estimating ${\hat{\lambda }}_{*}=Y_{*}/n_{*}$, one has the Poisson sampling error $SE_{P}\left(Y_{*}\right)=\sqrt{Y_{*}}$, or equivalently $SE_{P}\left(y_{*}\right)=\sqrt{Y_{*}}/n_{*}=\sqrt{y_{*}/n_{*}}$. However, this does ignore the overdispersion stemming from intra– and inter–individual variation. We know again from the theory of the simple exponential family that under the presence of dispersion, one has $\text{Var}\left(Y_{*}\right)=\phi {\text{SE}}_{P}^{2}\left(Y_{*}\right)$, and so $\text{SE}\left(y_{*}\right)=\sqrt{\hat{\phi }y_{*}/n_{*}}$ [32]. Summarizing, we get from (8)

$$\begin{array}{c} SE^{2}\left(x_{*}\right)=\frac{1}{{\hat{B}}^{2}}SE^{2}\left(\hat{A}\right)+\frac{{\left(y_{*}-\hat{A}\right)}^{2}}{{\hat{B}}^{4}}SE^{2}\left(\hat{B}\right)+\frac{1}{{\hat{B}}^{2}}\frac{\hat{\phi }y_{*}}{n_{*}}. \end{array}$$ (12)

It is noted once more that this approach is for situations in which, as is common for H2AX data, the focus information is provided by a single number per cell—the yield y_*_, that is the number of foci per cell over n_*_ cells. If full frequency distributions of focus counts are available, an alternative way of modelling their variability could be to estimate the scale or shape parameter of an appropriate two–parameter model (such as a negative binomial model). Some further comments in this respect are provided in the Discussion (Section 6).

### 4.2 Reference samples

The preceding discussion assumes that the calibration curve C is actually fit for purpose. While the standard errors mentioned above account for inter– and intra-individual variation as well as measurement and random error *with mean 0* around the calibration curve, they do not account for systematic deviations (bias) from this curve, for instance by different technology used in laboratories, different conventions of focus scoring by different scorers, etc. It has therefore been proposed in the literature to produce *reference samples* [13, 11]. These are samples irradiated at *known* doses and scored under the same conditions as the data N. We assume for ease of presentation that there are only two of such reference samples, taken at doses *x*_0_ = 0*Gy* and *x*_*r*_ = *rGy*, with resulting yields *y*_0_ and *y*_*r*_, where typically *r* = 1.5. (If more than one reference sample has been obtained for a given dose point, the resulting yields can be added so that one has again only one reference sample each.) We assume that *n*_0_ and *n*_*r*_ cells have been scored, respectively, and denote the information {*y*_0_, *n*_0_, *y*_*r*_, *n*_*r*_} by R.

It then needs to be worked out whether R is consistent with C. One approach is to compare *y*_0_ and *y*_1.5_ with prediction intervals (PI) around the calibration curve. There is no ultimate consensus in the statistical literature on how prediction intervals for quasi-Poisson regression models are to be obtained, but using the same line of thinking as above, it is plausible to find approximate prediction intervals for yield *y* at dose *x* (under the assumption that C is true) via

$$\begin{array}{c} \hat{A}+\hat{B}x\pm q_{\text{crit}}\sqrt{\frac{1}{n^{\prime }}\hat{\phi }\left(\hat{A}+\hat{B}x\right)}, \end{array}$$ (13)

where *n*′ ∈ {*n*_0_, *n*_*r*_} is the respective reference sample size, and *q*_crit_ is an appropriate critical value corresponding to the required level of confidence. Hence, a 95% PI for *y*_0_ would be approximated by $\hat{A}\pm 2\sqrt{\hat{\phi }\times \frac{\hat{A}}{n_{0}}}$, and similarly a 95% PI for *y*_*r*_ would be obtained by setting *x* = *x*_*r*_ and *n*′ = *n*_*r*_ in (13). It is impractical to compute the value $\hat{\phi }$ from the reference samples (one would require at least three reference yields to obtain an estimate via (5), which even then would be unreliable), so this value should be taken from ‘general consensus’ or the calibration curve.

**Fig 5:**
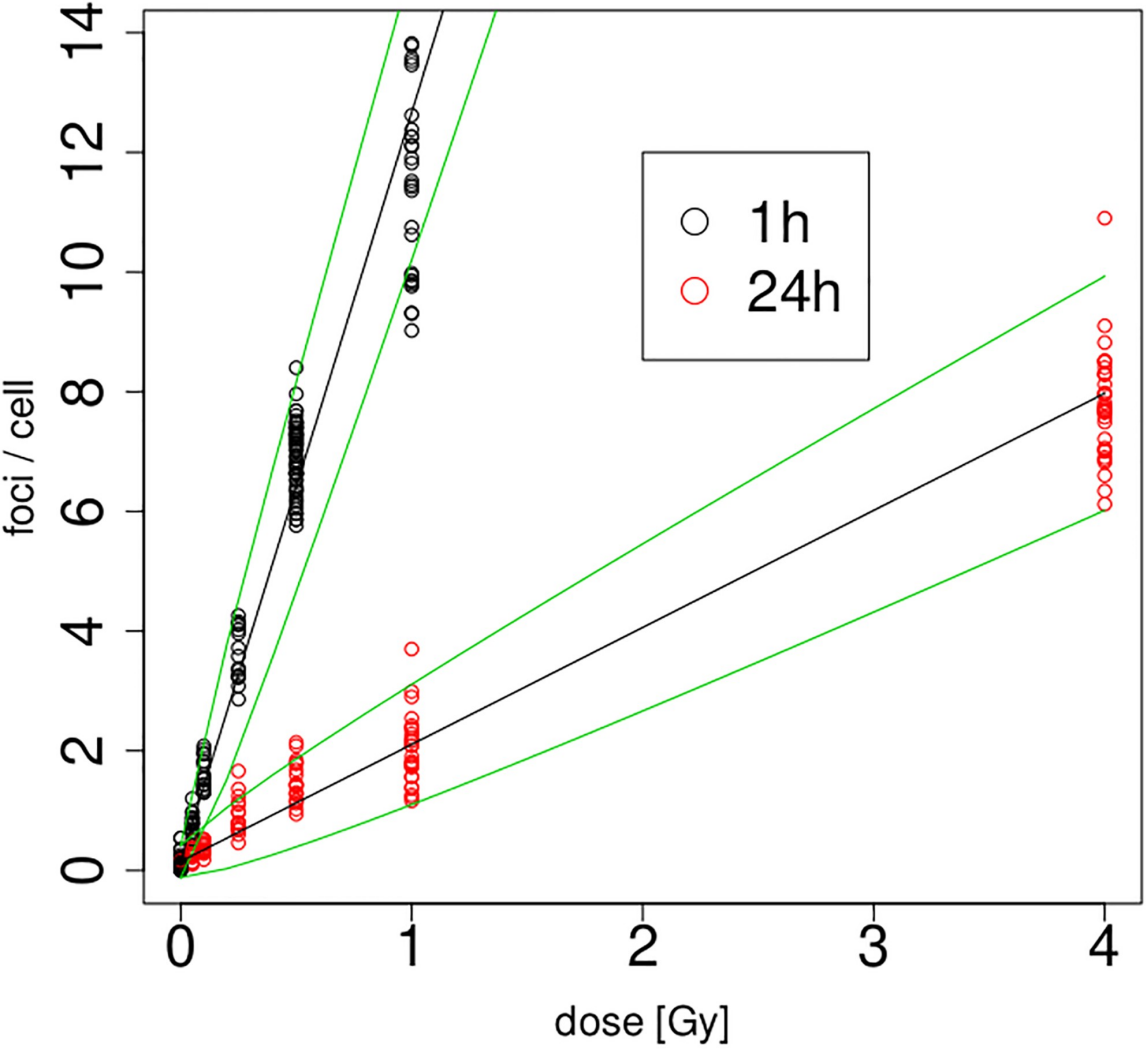
Linear calibration curves with approximate 95% prediction intervals.

Fig 5 gives approximate prediction intervals obtained in this way for the (jointly modelled) data from Section 2, using $\hat{\phi }=60$ and *n*_*r*_ = 500. If the reference yields falls outside the prediction intervals, the curve C is discarded. We observe that the width of the PI relative to the yield curve at *r* = 1.5 is about 30%, which corresponds to Public Health England’s internal working practice to discard a calibration curve if *y*_*r*_ deviates more than 30% from the curve. Note that there are two reference yields to check—the positive and the negative control—but only one decision to make, namely to discard or to accept the calibration curve. One will usually be more concerned with the slope, $\hat{B}$, as its misspecification can have very severe consequences. In contrast, the value of $\hat{A}$ will usually be small, as will be its standard error, and one would not want to throw away an established calibration curve for a minor mismatch of a negative control sample. Hence, for our purposes we decide to give more leeway to $\hat{A}$ than to $\hat{B}$, that is we set *q*_crit_ = 3 for the negative sample (*y*_0_) and *q*_crit_ = 2 for the positive sample (*y*_*r*_), and discard the entire calibration curve if any of them falls outside their respective prediction interval.

### 4.3 Reference curves

We will turn now to the question of how to proceed if the reference yields R disagree with the calibration curve C, that is if *y*_0_ or (more importantly) *y*_*r*_ fall outside their prediction intervals. Here we start with a trivial but important observation: A straight line is determined already by two points. Hence, the two reference points determine a calibration curve in their own right. Of course, the ‘reference curve’ parameters obtained this way will generally have larger variance than the calibration curve parameters, but, more importantly, they can reasonably assumed to be unbiased. The reference curve is then defined by

$$\begin{array}{c} E\left(y\right)=y_{0}+\frac{y_{r}-y_{0}}{r}x \end{array}$$ (14)

that is $E\left(y\right)=\tilde{A}+\tilde{B}x$ with $\tilde{A}=y_{0}$ and $\tilde{B}=\left(y_{r}-y_{0}\right)/r$. Standard errors of these parameters can be immediately obtained along previous lines as

$$\begin{array}{ccc} \text{SE}(\tilde{A}) & = & \text{SE}\left(y_{0}\right)=\sqrt{\phi \frac{y_{0}}{n_{0}}}; \end{array}$$ (15)

$$\begin{array}{ccc} \text{SE}(\tilde{B}) & = & \frac{1}{r}\sqrt{{\text{SE}}^{2}\left(y_{0}\right)+{\text{SE}}^{2}\left(y_{r}\right)}=\frac{\sqrt{\phi }}{r}\sqrt{\frac{y_{0}}{n_{0}}+\frac{y_{r}}{n_{r}}} \end{array}$$ (16)

where the quasi-Poisson assumption has been exploited to estimate the variance of focus counts per cell. As mentioned before, it is not practical to estimate *ϕ* from the reference data, so this value again has to come from the calibrative data or general consensus.

So, summarizing, we propose to initially check whether the reference samples are consistent with the calibration curve, and if so, use the calibration curve. If the reference sample is inconsistent with the calibration curve then the reference data can be used to obtain a ‘reference curve’, which still allows dose estimation, albeit with much higher uncertainties. These uncertainties will swamp the dose estimates on some occasions, but often they will still be precise enough to at least enable triage of the sample N under consideration. One could also contemplate using hybrid versions where the reference data are used to ‘update’ the calibration curve, either in a Bayesian or empirical Bayesian framework [9], or through appropriate weighting schemes, but such approaches are not considered herein.

If no reference data are available then the only option left is to use the available calibration curve uncontested, which will still allow the production of dose estimates. It is important that any software or output which reports estimates in such cases also gives a corresponding warning message (our web applet does so). The whole process is implemented in a web applet which is introduced in Section 5.2.

### 4.4 Reference sample ratios

In practice, it has been observed that reference sample yields tend to exceed the calibration curve yields. The reason for this is that reference samples are not subject to focus loss due to shipment. Hence, it can be argued that reference yields which fall below the calibration curve are suspicious either way, even if they fall within the range of the prediction interval (or a 30% range, for that purpose). It has been suggested [11] to multiply the dose estimate obtained from (7) with the *reference sample ratio* $\left(\hat{A}+\hat{B}x_{r}\right)/y_{r}$ to adjust for this effect. Mathematically, this amounts to pulling the calibration curve down so that it passes through $\hat{A}$ and (approximately) *y*_*r*_, where several variants of this scheme are possible. Individual laboratories will be best placed to decide whether such adjustments (which are, to repeat this, operating within the range of the sampling variation) are necessary. Our web applet does not make such adjustments.

## 5 Case study and web applet

### 5.1 Data analysis

We now apply the methodology introduced in Section 4 to a manually scored *γ*-H2AX foci data set obtained in an *in vitro* setting at Public Health England after irradiation of blood lymphocytes with 250kVp X-rays (with identical technical specifications as for the previous data set). Venous blood was taken with informed consent and the ethical approval of the West Midlands–Solihull Research Ethics Committee (REC 14/WM/1182). This is part of a much larger data set which also contained focus counts 4h after exposure, some measurements for doses at 4Gy, as well as partial exposure measurements which we have excluded from this discussion for different reasons. It leaves a total of 12 aggregated measurements, as displayed in Table 4. We split these into four observations to be used as reference samples (bold), and eight observations from which to estimate doses (and which are for this purpose assumed unknown).

**Table 4:** Data from Section 5.1. Rows in bold print are considered as reference information. The first column gives the true, known doses (which are only made use of for the reference samples). Dose estimates and standard errors are provided in the final two columns. Yields associated with a ◇ symbol were obtained by a different scorer, in the same laboratory.

*x*	time	*n*	*Y*	*y*	x_*_	SE(x_*_)
**0**	1h	200	68	0.34		
0	1h	200	143	0.72	0.08	0.18
0	1h	200	69	0.35	−0.04	0.14
**0**	24h	200	117	0.59		
0	24h	200	64	0.32	0.09	0.16
0	24h	200	^◇^16	^◇^0.08	^◇^-0.04	^◇^0.08
0.75	1h	53	293	5.53	1.67	0.94
0.75	24h	200	153	0.77	0.32	0.24
**1.5**	1h	200	1004	5.02		
**1.5**	24h	200	583	2.92		
3	1h	200	1305	6.53	2.00	0.72
3	24h	200	^◇^552	^◇^2.76	^◇^1.33	^◇^0.46

It is noted that the data labelled with a ◇ were collected through a different scorer, and that the 0Gy data still carry a time label of 1h and 24h which means that this time span ‘was waited after not exposing the data to radiation’. We mentioned before that in this case the time span is essentially meaningless, and hence we use the first of each of the three measurements to construct a *joint* reference sample. However, when estimating the dose of the non–reference samples we still assume that these yields are allocated to the respective 1h or 24h curves, since in this case we do not know that the true dose is 0Gy and so one still needs to make use of a full calibration curve.

We assume for this analysis that the available calibration curves are the ones provided in the bottom of Table 2, with $\hat{\phi }=60$. The next step is to check for the compatibility of the reference information with the calibration curve. For the control yield at dose zero, we have *y*_0_ = (68 + 117)/400 = 0.4625. This is compared with a prediction interval $0.150\pm 3\sqrt{60\times 0.150/400}=\left[-0.3,0.6\right]$ so we accept the value $\hat{A}$ of the calibration curve. For the positive control samples at 1.5Gy, one has at 1h the reference yield of 5.02 which is outside the 95% prediction interval

$$\begin{array}{c} \begin{array}{cc} & 0.150+12.518\times 1.5\pm 2\times \sqrt{60\times (0.150+1.5\times 12.518)/200}\\ & =[14.2,23.7]. \end{array} \end{array}$$ (17)

So, the 1h curve is rejected as a whole, and replaced by $\tilde{A}=0.4625$ with $\text{SE}\left(\tilde{A}\right)=\sqrt{60\times 0.4625/400}=0.263$, as well as $\tilde{B}=\left(5.02-0.4625\right)/1.5=3.038$ with $\text{SE}\left(\tilde{B}\right)=\frac{\sqrt{60}}{1.5}\sqrt{\frac{0.4525}{400}+\frac{5.02}{200}}=0.837$. At 24h, we have the reference yield of 2.92 which lies just in the middle of the prediction interval [1.160, 5.007] which is centered at the fitted value of 0.150 + 1.5 × 1.956 = 3.084. So, here the curve is clearly accepted. We proceed now with estimating the remaining doses. For the 1h data, we have the yields of y_*_ = 0.72, 0.35, 5.53, and 6.53 available, and we need to use the reference curve 0.4625 + 3.038*x* to estimate the doses. For the 24h data, taking the results by the second scorer aside for a moment, we have the yields of y_*_ = 0.32 and 0.77 available, and we can use the calibration curve 0.150 + 1.956*x*, with appropriate standard errors as reported earlier. The results after application of Eqs (7) and (12) are provided in the columns for x_*_ and SE(x_*_) in Table 4. One can see from this that—as expected—most of the dose estimates are not very precise, but all of them are within two standard errors of the true values, where it is noted that the standard errors tend to be larger when the reference curve has been used (1h) rather than the calibration curve (24h). Despite their imprecision, all dose estimates appear useful enough to enable triage.

The data from the second scorer are more challenging to deal with. The change of scorer implies a change in experimental conditions so that the previous reference data cannot be used to judge the adequacy of the calibration curve. Since no reference sample is available for this scorer, all what can be done is to use the uncontested calibration curve, and produce a corresponding warning message, which we again highlight by a ◇ in Table 4. It is clear that in this case one of the two estimates turned out to be reasonable but the other one was compromised by the lack of adequate reference data, being more than 3 standard errors away from the true value.

**Fig 6:**
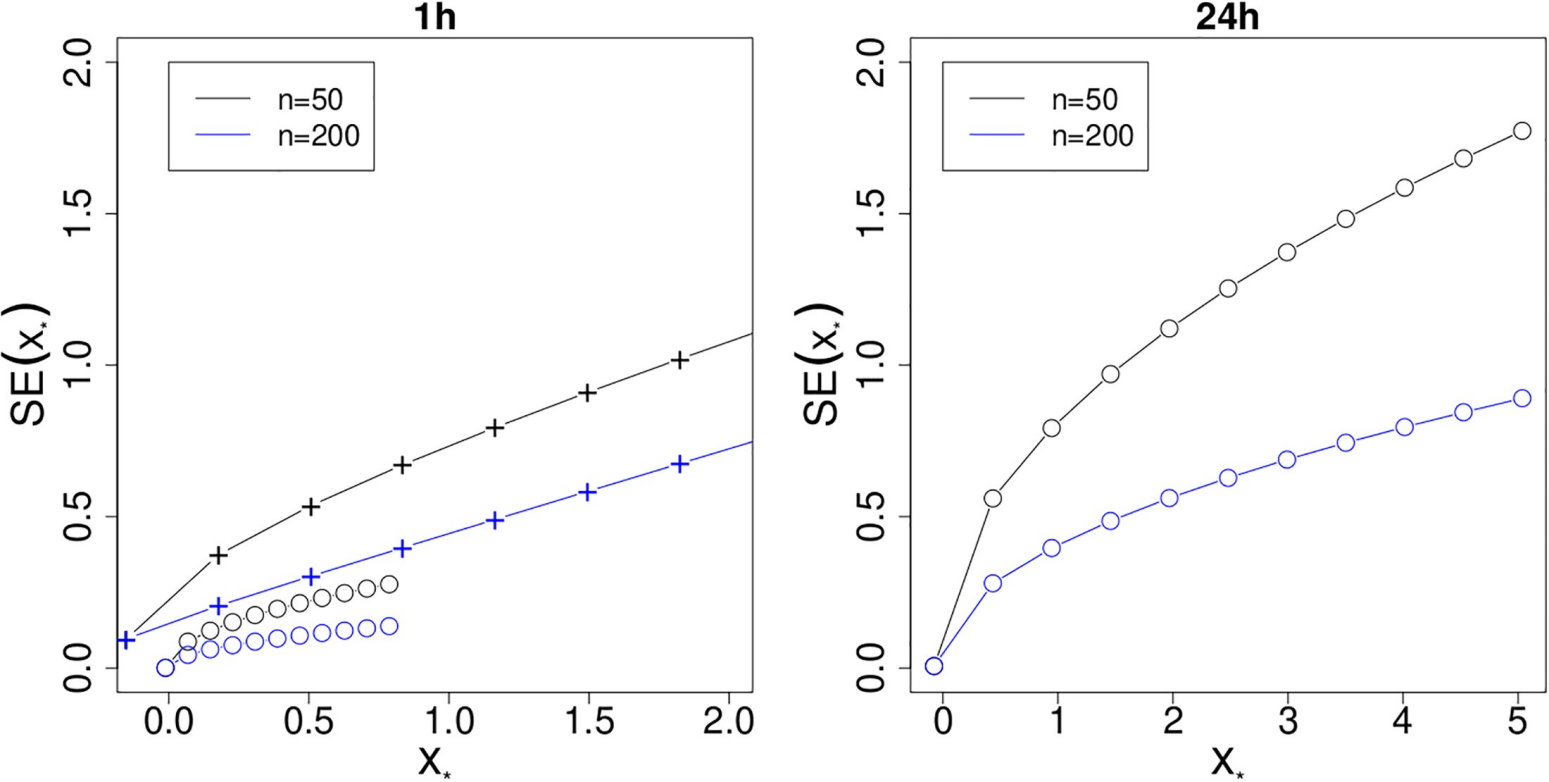
Plots of standard errors, SE(x_*_), versus dose estimates, x_*_, for 1h (left) or 24h (right) after exposure, for n_*_ = 50 (black) or 200 (blue) available cells, respectively. Measurements symbolized through circles ∘ assume that the given calibration curve is true, while those displayed through a + symbol assume that it is rejected and hence a reference curve is used. The yields corresponding to the circles/crosses along the curves are given by 0, 1, 2, …, 10.

Finally, we compute dose estimates and their variances for a grid of yields in order to get a more complete picture of the variability in this process. Initially, let us assume the 1h calibration curve to be ‘correct’, and take the grid of yields from 0 to 10 with step size 1. Then, we compute dose estimates and variances according to (7) and (12), assuming measurements from n_*_ = 50 and 200 cells, respectively. The results are visualized through the black and blue curves (connected by circles) in Fig 6 (left). Recall firstly that dose estimates do not depend on n_*_, so each two circles or crosses are always perfectly vertically aligned. Now, if the 1h calibration curve *was* adequate for our data, we would see that the standard errors of dose estimates were consistently below 0.3Gy. But, of course, this calibration curve was not validated and it is also clear from the figure that this ‘wrong’ curve would lead to tiny dose estimates. If the reference curve is used instead, the dose estimates and their standard errors are inflated considerably (curves connected by + symbols). The right hand panel shows the standard errors arising from the (validated) 24h calibration curve, using the same grid of yields as before. We see that standard errors can come close to 1Gy for n_*_ = 200 and 2Gy for n_*_ = 50.

### 5.2 Web applet DoseEstimateH2AX

A web applet is available at http://asapps.bcamath.org:5054/ which allows one to perform biodosimetric dose estimations from H2AX data, following the methodology proposed in Section 3. This applet has been developed using Shiny RStudio technology [33]. This applet can be used to reproduce the examples from this manuscript, but of course it can also be applied to any other data set (calibration data, reference samples, and customer yields), and it is hoped that laboratories and public health institutions will find this tool useful.

In its current version, only two H2AX calibration curves, namely PHE’s 1h and 24h curves according to the bottom part of Table 2, are implemented as ‘built–in’ curves. However, usually laboratories will want to use their own calibration curves, and it is in fact recommended to do so. The applet allows for entering such calibration data. In either case, whether built–in or user–supplied, the calibration curve should be validated via reference samples (one control and another irradiated) which can be entered by the user into the system. If the calibration curve is validated then it is used in the following dose estimation step, but if it is not validated then the reference samples are used to construct a new calibration curve as previously explained. If no reference samples are provided then dose estimates will still be produced, accompanied by adequate warning messages.

In the event the user introduces the fitted calibration curve parameters but does not provide their respective standard errors, they are taken as 0 in expression (6). Analogously, if the dispersion index is not introduced by the user, this is taken as 60. Warning messages are displayed accordingly.

**Fig 7:**
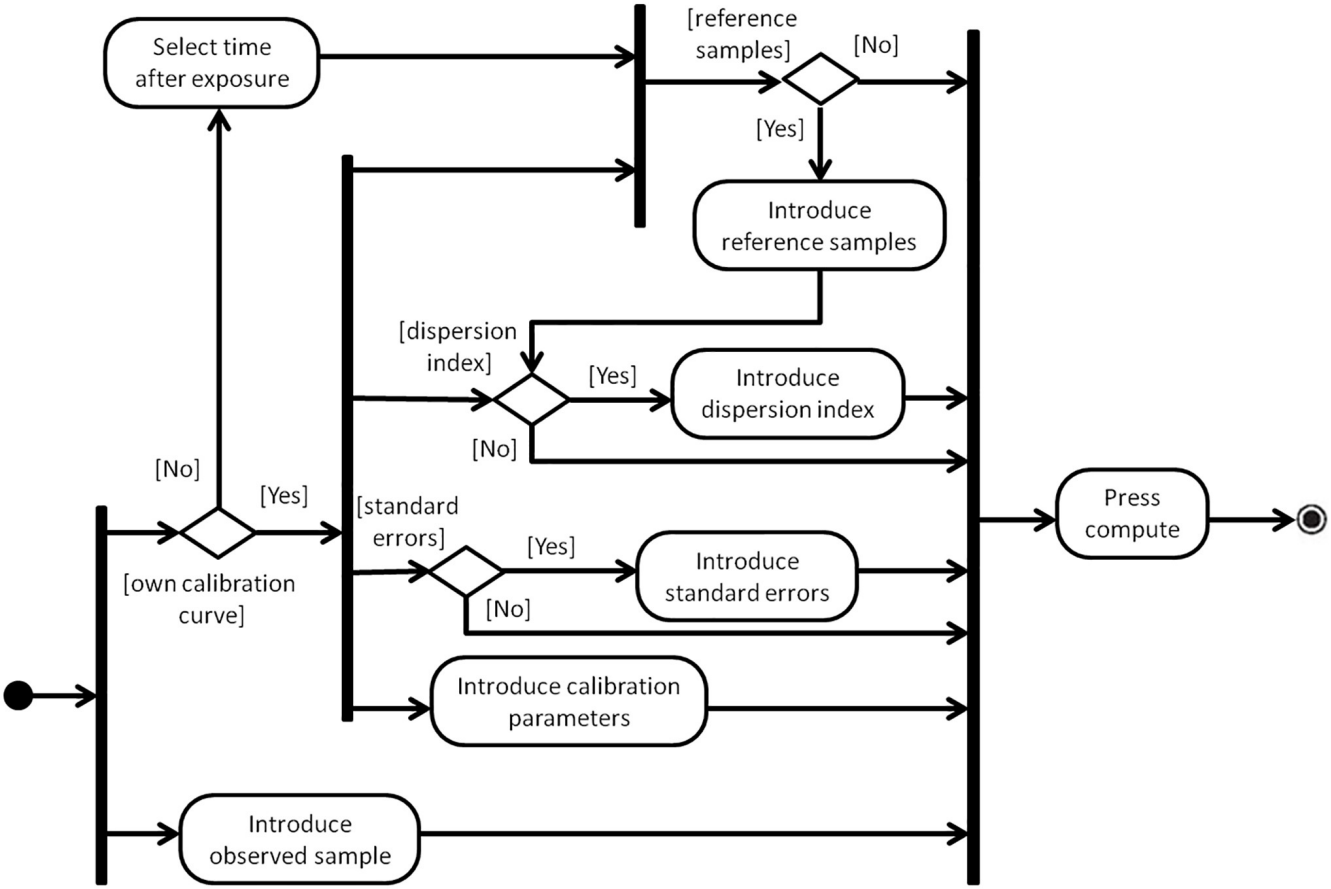
Activity diagram for web applet DoseEstimateH2AX.

Fig 7 shows the activity diagram of the applet, representing the different paths through which a user can get a H2AX based biodosimetric dose estimation. The authors encourage the users to give feedback for updating the applet to improve their user experiences (the applet’s ‘Information’ tab describes how to do this). Through the same route the users are also encouraged to make their calibration data available to the developers so that they can be included into later versions of the applet and thus increase its capabilities.

**Fig 8:**
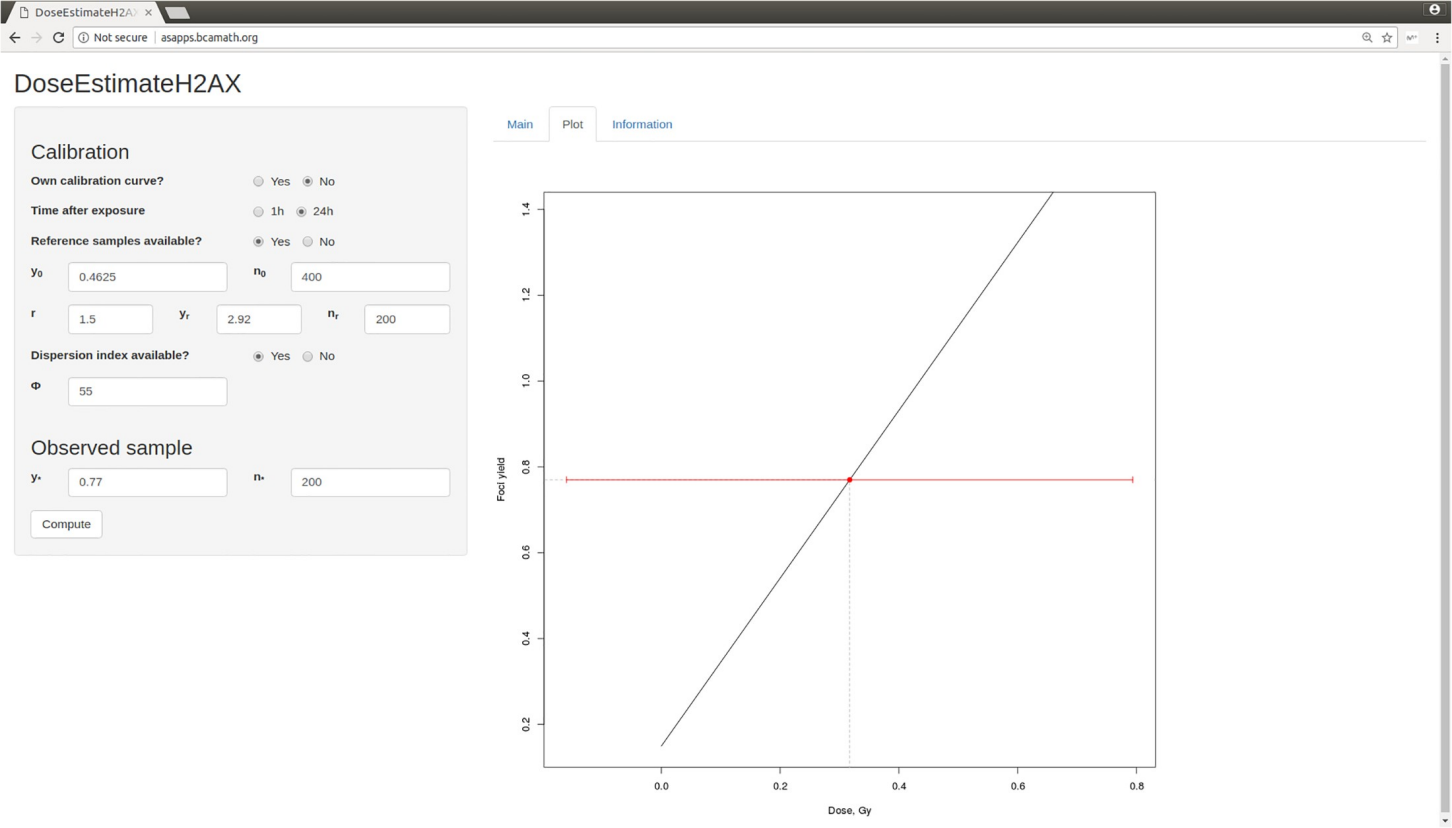
Illustration of web applet using the 24h curve with y_*_ = 0.77 and n_*_ = 200, as well as reference samples according to Table 4. The point estimate x_*_ and the interval estimate x_*_ ± 1.96SE(x_*_) of dose are highlighted through a red dot and line, respectively. Numerical output can be obtained by clicking on the “Main” button, see the Tutorial in S1 File for details.

An example for the use and graphical output of this applet, for the case of the 24h calibration curve as elaborated in detail in Subsection 5.1, is provided in Fig 8. A tutorial on how to use this applet, which contains several further examples addressing different scenarios, is provided in S1 File.

## 6 Discussion

For retrospective dosimetry using the *γ*–H2AX assay, ready–to–use methodology and software (in form of a publicly available web applet) has been developed for the estimation of radiation doses. As part of its workflow, the methodology checks for validity of the supplied calibration curve and produces a substitute calibration curve from the reference samples otherwise, hence ensuring that in either case the point and interval estimates of dose are ‘correct’, with variability bounds not larger than necessary. This new methodology takes the specific character of H2AX data into account, rather than relying on dicentric techniques which turned out to be not fully fit for purpose in this context.

A specific feature of H2AX which has been frequently reported is the poor reproducibility of experimental results [19, 21, 27]. There are several layers of this reproducibility problem. In the outer layer, one could ask whether different laboratories are able to reproduce each other’s results. A systematic study in this direction was carried out in the framework of the RENEB network [19]. The key question that this boils down to is whether the laboratories use a valid calibration curve. Hence, it is essential for this purpose that laboratories use appropriate reference samples to validate their curves. Our work in subsections 4.2 and 4.3 provides methodology to carry out this check, and also provides a way forward in case the calibration curve could not be validated. To some extent, this step will also account for shipment effects, or for situations where the ‘time after exposure’ is incorrectly assessed as these, when significant, will cause the calibration curve to be rejected.

Somewhat more interesting (and more challenging) is the problem of ‘internal reproducibility’, which we define as the ability of a specific laboratory, having scored foci from a given blood sample, to reproduce their own result. There are two types of variation which impact adversely on reproducibility in this sense; namely variation due to the focus scoring process, and variation related to the individual. For the former, we need to distinguish further. Firstly, there are physical and chemical aspects relating to the measurement process itself (Moquet et al. [19] cite staining quality [20] and microscope settings [16]). This variation could be minor and random (in which case it gets absorbed by the overdispersion), but also substantial and systematic. Secondly, visual (manual) H2AX focus scoring is difficult even for trained technicians, and can lead to ‘dramatic variations’ [21]. We have ourselves observed (in the second data set introduced in this paper) systematic and significant differences in the amount of foci scored by different scorers. Such variation is of discrete (in our case binary) nature and will not necessarily be captured by allowing for overdispersion. Under our approach, systematic effects of this or other type would be identified in the validation process of the calibration curve: If the reference samples produced by the scorer are not compatible with the calibration curve, then the curve will be discarded and replaced by a new one obtained from the reference samples. It is, therefore, paramount that the reference samples are obtained by the same scorer as the one who will actually score the sample of interest—otherwise the procedure will break. Concerning variation due to the individual, this can be distinguished into inter– and intra– individual variation, as carefully elaborated on in Section 2. The latter one especially is of some conceptual concern, since it implies that even if one could control all previously mentioned types of variation, and even if samples are collected from the same individual, the results are still not reproducible. But, as we have demonstrated in this manuscript, this lack of reproducibility is in fact nothing other than high variability, which is captured (along with the inter–individual variation) through the dispersion parameter. Hence, we do believe that our approach addresses the main reproducibility issues one way or the other.

One central building block of our approach is the estimation of the dispersion index, which may be refined in further studies. The recommended value of $\hat{\phi }=60$ reflects the ‘maximum’ dispersion under *manual* focus scoring. It has been noted that automated scoring techniques increase the overdispersion in the scored counts [13]. Hence, it would appear plausible that the dispersion estimate under automated scoring needs to be considerably higher. A related question is whether partial body exposure can be detected from the level of overdispersion. Rothkamm et al. [13] suggest that this may still be possible under manual scoring but will become impractical under automated scoring due to the extreme overdispersion which is then encountered. The present study sheds some slight doubt on the ability to detect partial exposure even under manual scoring, since also in this case the focus counts under homogeneous exposure are far from equidispersed. Assuming that partial body exposure can be detected, the next question is to estimate the dose and exposure fraction. An instance of a successful application of Dolphin’s contaminated Poisson method [6] in this context has been presented in [17]. While partial body exposure is beyond the scope of our work, further research on this matter still appears desirable, since real life exposures are virtually always heterogeneous.

Readers who are familiar with the the literature on radiation dose estimation through the dicentric assay will be used to the presentation of the data in the form of frequency tables, which give, for each calibration dose point, the full count distribution of observed abberations [6, 8, 9, 34]. Such raw frequency data have been, originally, also available in our context, and in principle they could be similarly displayed. However, note that the maximal counts for H2AX data are usually on a considerably larger level (say 20–24) compared to dicentric data, where this maximum is typically 6 or 7 (the maximum number of biologically possible dicentric chromosomes in a cell is 23; for *γ*−H2AX foci there is no such limit).

In the methodology outlined in Section 3.1, we have aggregated the individual cell-by-cell counts to obtain counts per cell, or yields, *y*_*ij*_. This procedure implies an information loss as compared to the use of full frequency distributions. However, the yields constitute the sufficient statistic for the estimation of the Poisson mean function *μ*_*i*_, and this property does carry over to the quasi–Poisson case. So, any loss of information would only come into play as far as as variance, or dispersion, estimates are concerned. We are able to estimate the dispersion parameter consistently via (5) from the fitted Poisson model (which used only the yields). However, an alternative way to allow for overdispersion would be the use of a two-parameter model such as the negative binomial model [35], for which one would indeed need the full frequency distribution in order to estimate its shape parameter (which determines the dispersion), see [36] for the explicit formula of this estimator. The relative advantages and disadvantages of quasi-Poisson and negative binomial modelling strategies have been discussed in [37]. To our knowledge, analyzing frequency distribution data in a *γ*−H2AX context has not been systematically attempted yet, and we also do not consider this question further in this paper.

We believe that a very attractive direction forward for the field of biodosimetry is the combination of biomarkers. The idea would be to use a quick and cheap biomarker with potentially high but quantifiable variance, for instance one based on gene expressions [38] or proteins, for the initial triage step. Depending on the outcome of the triage, a more elaborated biomarker such as the dicentric assay can be subsequently carried out. If a Bayesian approach [9] is adopted for the dose estimation in the second step, then this idea appears particularly appealing as the triage outcome could be used directly as a prior in the Bayesian analysis.

Perhaps it is also time to reconsider the fixation of the community on always estimating *dose*. There appears to be no immovable reason why triage would always need to go via a dose estimate. Assuming that a well–defined set of triage categories can be specified, these could be linked with much simpler statistical methodology (effectively, multi–category logistic regression) directly to the *γ*-H2AX focus counts, which would circumvent the need for the complicated inverse regression techniques entirely. Of course, fitting such models would require the production of appropriate training data to start with. We leave such thoughts to further research.

## Supporting information

S1 File DoseEstimateH2AX tutorial. (PDF)

S2 File PHE calibration data (1h and 24h) in .xls and .dat format. (ZIP)
