# Supplementary material for: A statistical framework for radiation dose estimation with uncertainty quantification from the γ-H2AX assay
Source: PLoS One. 2018 Nov 28;13(11):e0207464. doi: 10.1371/journal.pone.0207464 (PMC6261578; doi:10.1371/journal.pone.0207464)

DoseEstimateH2AX tutorial (supplementary material of Einbeck *et al.* 2018)

This text is intended to assist in the usage of the applet DoseEstimateH2AX. This applet is available in the following links:

- http://asapps.bcamath.org:5054/
- https://manu2h.shinyapps.io/DoseEstimateH2AX/

For any kind of enquiry related with this applet, do not hesitate to contact Dr. Manuel Higueras or Dr. Jochen Einbeck.

1. A first look at DoseEstimateH2AX

This applet has a basic structure with a side panel (on the left) which focuses on the input interactions and a main panel (on the right) which is devoted to the results. This main panel is arranged in different tabs. The figure below explains this simple structure.

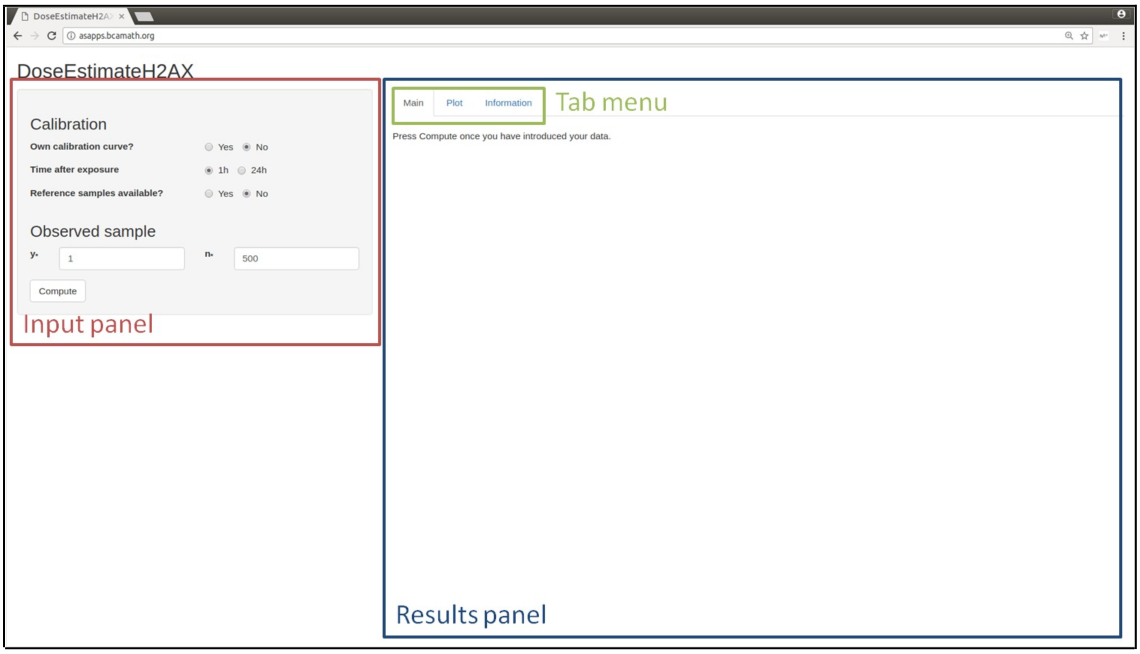

The 'Main' tab will display the fitted calibration parameters, the dose estimate and its statistical uncertainties, and warning messages if appropriate. The 'Plot' tab will display a graphical representation of the output, with a point and interval estimate of dose.

In the 'Information' tab, relevant updated information for using this applet will be provided.

2. Different workflows when using DoseEstimateH2AX

This applet allows the user to perform dose estimations based on H2AX assays. The system contains two built-in calibration curves, both have been constructed in Public Health England's Cytogenetics Group laboratory from X-rays irradiations, one for 1 hour and the other for 24 hours after exposure.

Depending on the information the user provides to the system, one can identify the following main workflows when dealing with this applet. They are described in the following items **a.**, **b.** and **c.**

a. The user does not provide any calibration information

In this case the user only provides the information about the observed sample, *i.e.* the frequency of H2AX per cell (field 'y_*') and the sample size (field 'n_*') and the time after exposure. The dose estimation is then carried out using one of the built-in calibration curves, depending on the selection of time after exposure. This choice displays a warning indicating that the built-in curve has not been validated for the user's lab samples.

The two following illustrations show the appearance of the applet when the user asks for dose estimation respectively 1 and 24 hours after exposure. The samples introduced correspond to both 0.75 Gy irradiated samples in Table 4 of the main text.

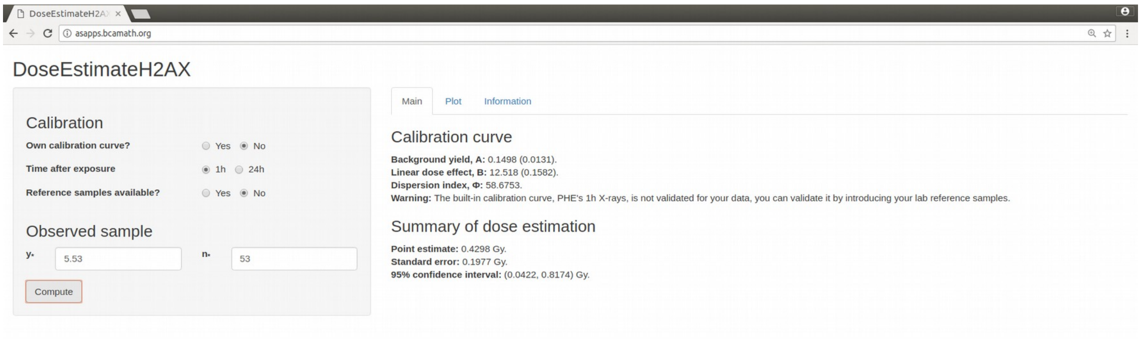

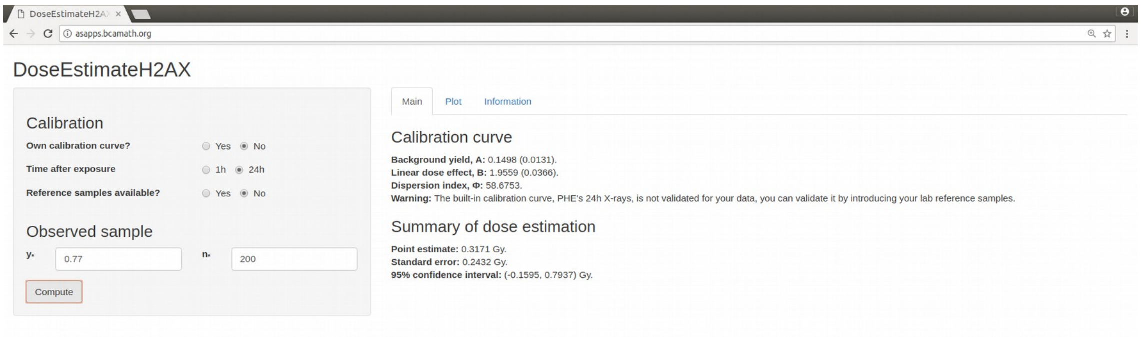

b. The user provides a calibration curve and does not provide reference samples

If apart from providing the information of an observed sample, the user also provides the information about the calibration curve, the user must select 'Yes' in the 'Own calibration curve?' field. The system lets the user insert the fitted calibration coefficients: the background yield (field 'A') and the linear dose effect (field 'B'). The system also lets the user to provide the standard errors of the fitted parameters and the quasi-Poisson model dispersion index once the user selects 'Yes' in the corresponding fields ('Standard errors available?' and/or 'Dispersion index available?', respectively).

If the user does not provide the calibration standard errors, these are taken as 0 for calculating the dose estimation standard error. If the user does not provide a dispersion index, this is taken as 60 for the same aim. In these cases, a warning indicating the values taken is displayed.

If available, the standard errors (under the Poisson assumption) of the background yield and the linear dose effect are inserted respectively in the '$SE_P(A)$' and '$SE_P(B)$' fields. The dispersion index is inserted in the 'φ' field.

The two following illustrations show the appearance of the applet when the user asks for dose estimation providing an artificial calibration curve, 0.15+12D. In the first illustration only the dispersion index is provided, and in the second one also the standard errors of the calibration parameters. The sample introduced in both illustrations corresponds to the 1 hour 0.75 Gy irradiated sample in Table 4.

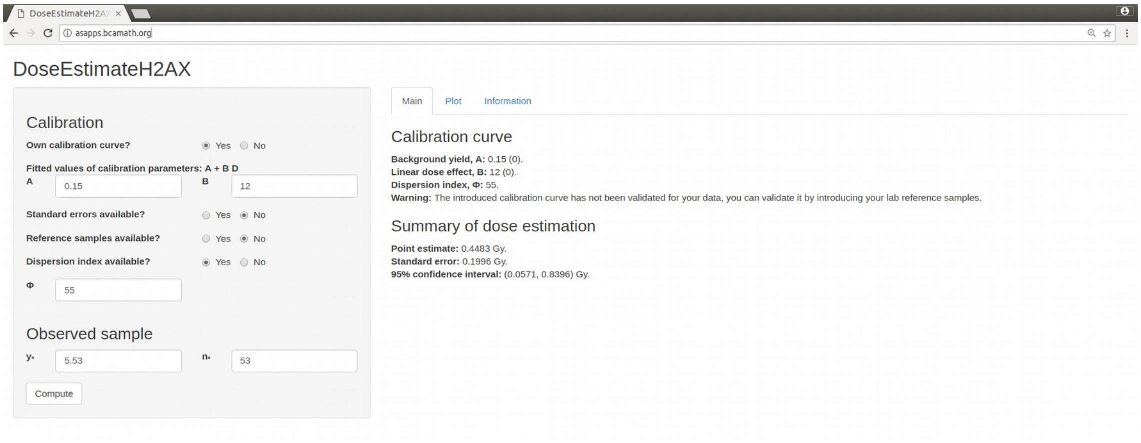

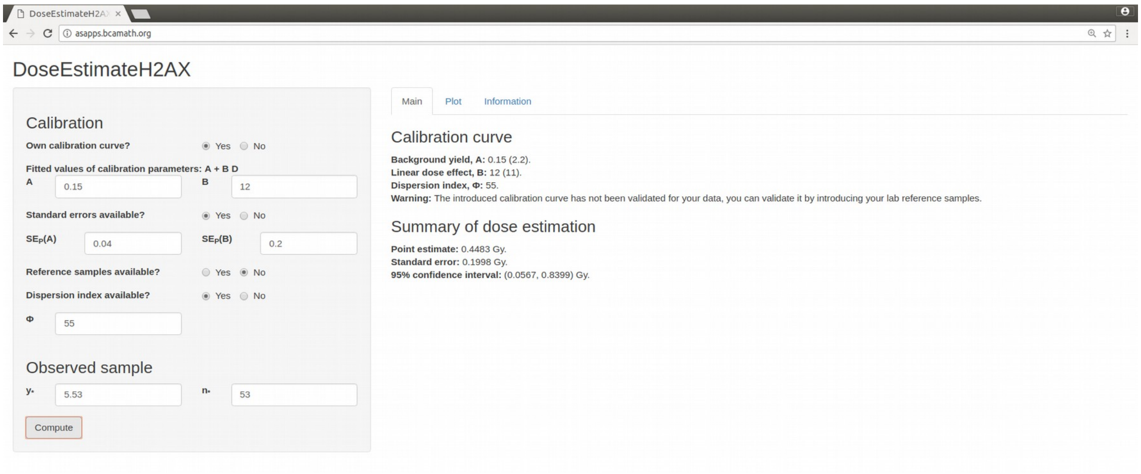

c. The user provides reference samples

The user has the choice to validate the used calibration curve (whether built-in or user-supplied) by introducing reference samples from their laboratory. For validating a built-in curve, the user has to select the time after exposure (to select the curve to be validated) and select 'Yes' in the 'Reference samples available?' field.

The system allows the introduction of two reference samples, one control and another irradiated. To introduce the control sample the user must introduce its yield of H2AX and sample size (fields 'y_0' and 'n_0' respectively). To introduce the irradiated sample, the user must introduce its irradiated dose, yield of H2AX and sample size (fields 'r', 'y_r' and 'n_r' respectively). If the built-in curve is validated (the conditions are explained in the main text of the publication), the dose estimation is performed with a message indicating this validation. If the calibration curve is not validated, a calibration dose-response curve is created from the reference samples and used for the dose estimation accompanied by a message indicating this situation.

For the built-in curves, the dispersion parameter for defining the prediction intervals is the fitted one from the curve. The user can introduce a dispersion parameter value

which will be applied if the reference samples do not validate the curve, and if not introduced this will be taken as the fitted one from the built-in curve.

For introduced calibration curves jointly with reference samples, the introduced dispersion index is used for the prediction intervals and for the calibration curves (the original if validated or the one based on the reference samples). If the dispersion index is not introduced, this is taken as 60.

The user can provide a dispersion parameter to be applied for the dose estimation uncertainties in case the reference samples do not validate the built-in curve or the introduced curve. If no dispersion parameter value is provided, this is taken as 60. This definition of the dispersion parameter value is the same as explained in the previous item (**b.**).

The two following illustrations show the appearance of the applet when the user asks for dose estimation validating respectively the 1 and 24 hours built-in curves. These two examples represent the dose estimation of both 0.75Gy irradiated samples from Table 4 of the main text, with the reference samples introduced as explained in Section 5.1 of the main text.

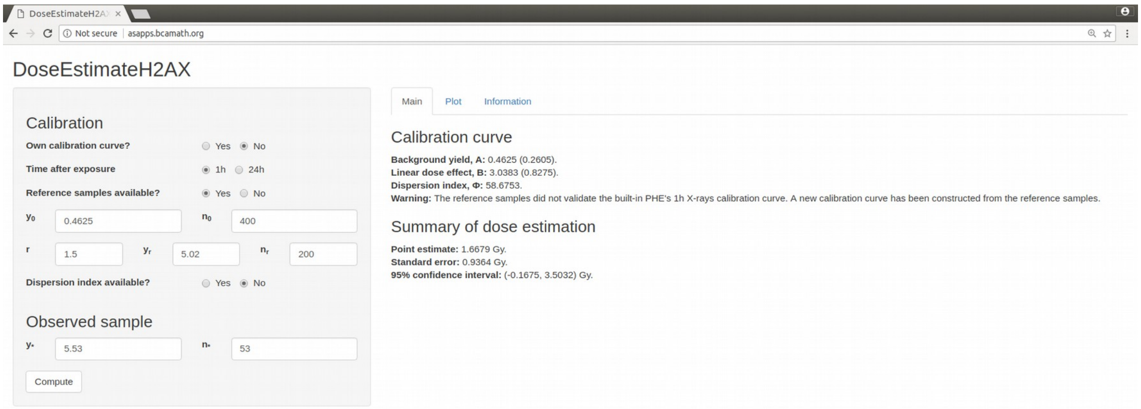

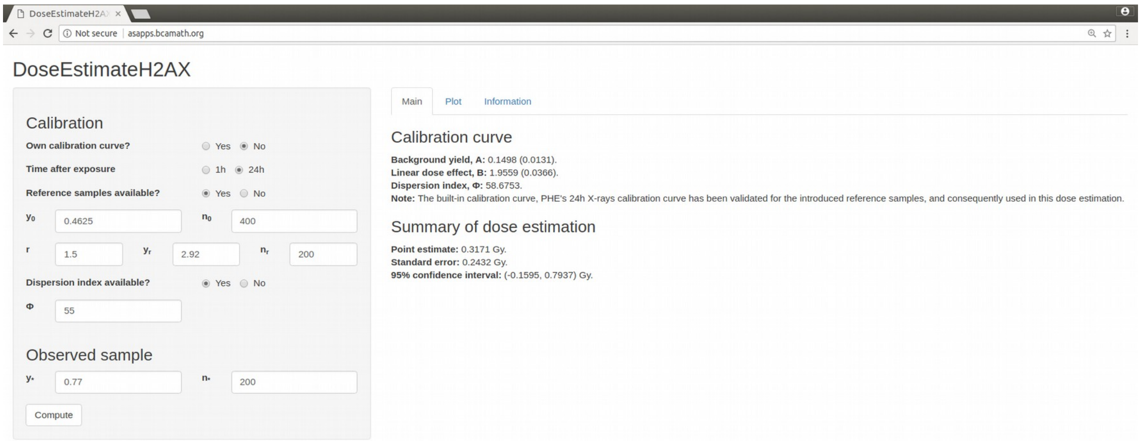

3. Graphical output

Once the dose estimation is performed, the 'Plot' tab shows a graph which represents graphically the dose estimation, visualizing the calibration curve, the point estimation and the 95% CI of the dose estimation.

The next illustration shows a brief description of the plot generated in the previous illustrated example.

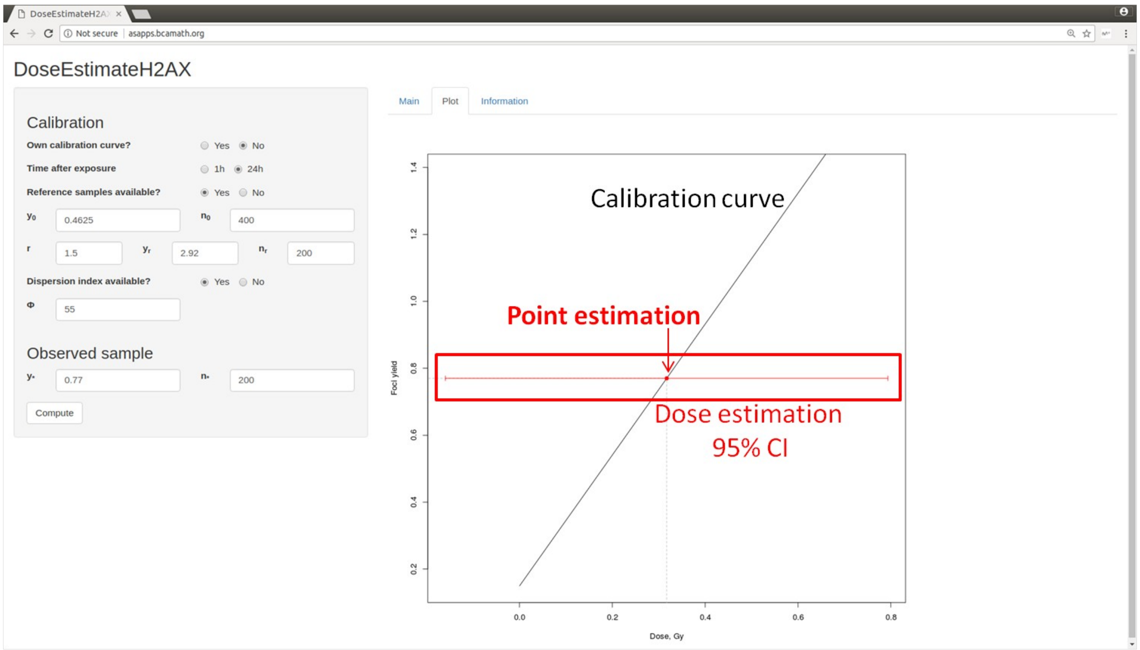

4. Enhancement in version 1.1: download reports

In DoseEstimateH2AX version 1.1, updated in the above URLs, the user has the choice of downloading an HTML report with the same structure of the results shown in the "Main" tab of the applet. The next illustration shows the appearance of the applet when the user can request the download of the report.

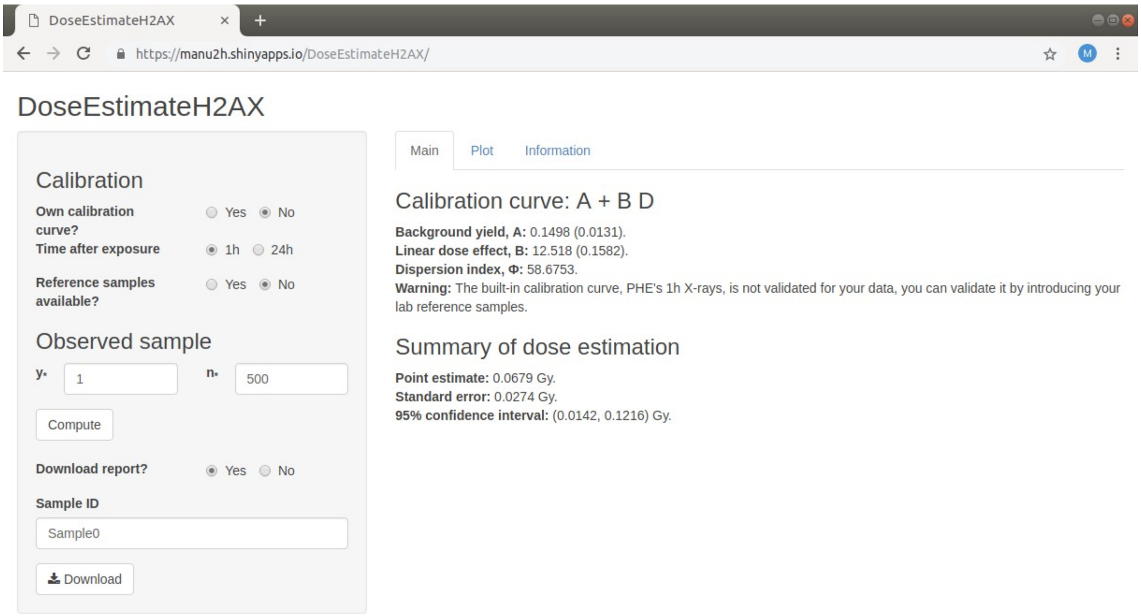

Supplement: S1 File — (PDF) [file pone.0207464.s001.pdf]
